# UNDERSTANDING HEARING LOSS AMONG COMMUNITY-DWELLING OLDER ADULTS FROM AN ENGAGING WITH AGING PERSPECTIVE

**DOI:** 10.1093/geroni/igad104.3029

**Published:** 2023-12-21

**Authors:** Shaoqing Ge, Kuan-Ching Wu, Jessica Jin, Suah Park, Shao-Yun Chien, Janet Primomo, Basia Belza

**Affiliations:** University of Texas at Austin School of Nursing, Austin, Texas, United States; University of Washington, Seattle, Washington, United States; University of Washington, Seattle, Washington, United States; University of Washington School of Nursing, Seattle, Washington, United States; University of Washington, Seattle, Washington, United States; University of Washington School of Nursing, Seattle, Washington, United States; University of Washington, Seattle, Washington, United States

## Abstract

Engaging with Aging is an emerging framework proposed by Carnevali which provides a new lens to understand an active, conscious daily living process of coping with age-related changes (ARCs) taken on by older adults. The study aims were to 1) describe the challenges related to hearing loss experienced by community-dwelling older adults, especially during COVID-19; 2) identify the strategies and resources used by older adults to accommodate the daily living challenges caused by hearing loss. We conducted semi-structured interviews with 29 participants aged 64 to 98 online due to COVID-19 restrictions. We used a virtual card sort to assist with data gathering. Hearing loss was mentioned by 18 participants (62%) and their corresponding adaptations were discussed. We found that older adults linked their adaptations to hearing loss based on their changing hearing capacities and needs. Commonly used adaptations included lip reading, volume adjustment, and wearing hearing aids. The challenges caused by COVID-19 in implementing the adaptations were also discussed (e.g., increased difficulty in understanding others due to mask-wearing). While most participants reported making adaptations to hearing loss, some mentioned that hearing loss was “no biggie” to them so no adaptations were needed. This study substantiates the EWA framework by showing the commonality among older adults in linking hearing loss, as one of the common ARCs, with corresponding adaptations. Implications for clinicians and researchers include using EWA to help older adults identify personalized solutions that fit their hearing capacity.

